# Case report: MRI findings of acute uremic encephalopathy in a 1-year-old boy

**DOI:** 10.1259/bjrcr.20210057

**Published:** 2021-05-14

**Authors:** Amar Ajay Chotai, Dipayan Mitra

**Affiliations:** 1 Department of Neuroradiology, Royal Victoria Infirmary, Newcastle-upon-Tyne, United Kingdom

## Abstract

We present a 1-year-old boy who presented to the emergency department with a 7-day history of diarrhoea and vomiting. The initial renal function profile demonstrated a urea of 55 mmol l^−1^ (normal range between 5 and 20 mmol l^−1^), creatinine 695 micromol/L (normal range between 62–106 micromol/L) and potassium 9.1 mmol l^−1^ (normal range between 3.5–5.0 mmol l^−1^), with a profound metabolic acidosis. Upon examination, there were no significant findings, specifically no neurological abnormality.

He was prescribed back-to-back Salbutamol nebulisers, to increase the shift of extracellular potassium into the intracellular space, followed by i.v. calcium gluconate, with some improvement in potassium levels. A further 5 mmol of sodium bicarbonate was given, as well as a stat dose of 1 mg/kg furosemide, and per rectal calcium resonium. He was then commenced on an infusion with 10% dextrose with insulin.

He was subsequently found to be in urinary retention and a catheter was inserted, which drained 1700 ml.

A subsequent renal function profile, 24 hours after admission, demonstrated improvement with urea 39 mmol l^−1^, creatinine 300 micromol/L and potassium 3.0 mEq/L.

## Investigations

**Figure 1.**
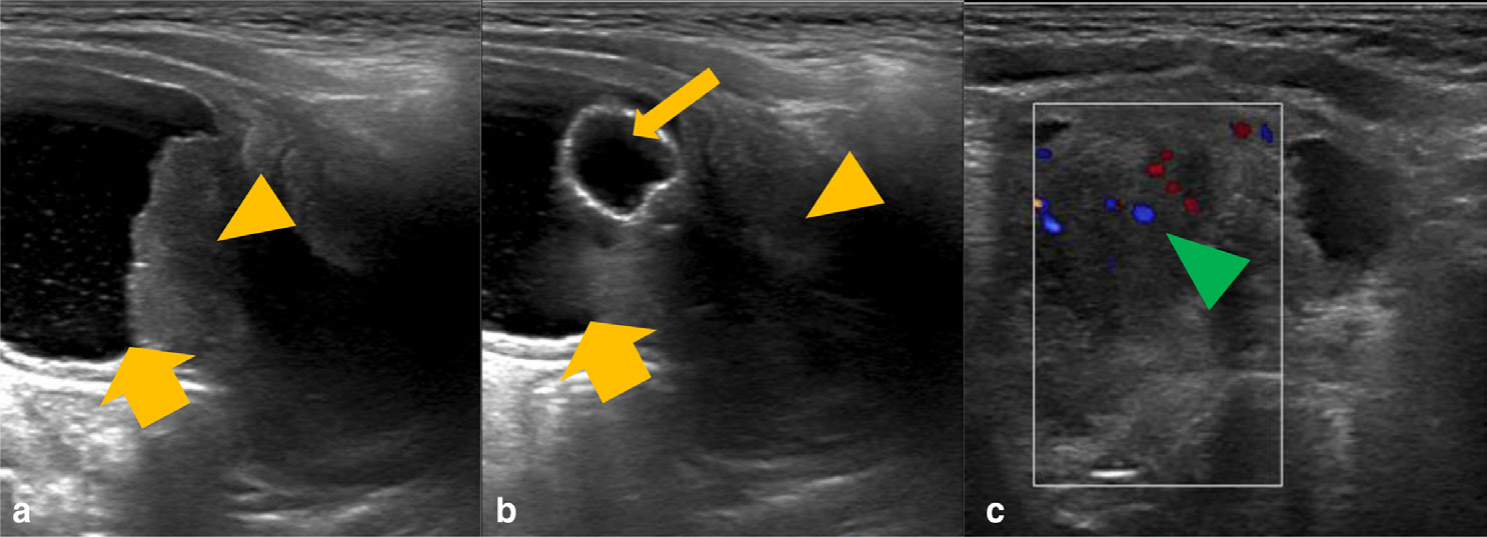
Grayscale ultrasound images of the bladder and prostate gland (**A and B**) with further colour doppler ultrasound (**C**). There is a heterogeneous mass in the expected location of the prostate gland (orange arrowhead), which is invaginating into the urinary bladder base (thick orange arrow). There is evidence of increased internal vascularity (green arrowhead). The urinary catheter courses through the mass and the balloon is within the urinary bladder (thin orange arrow).

The child had an initial ultrasound examination of the renal tract, demonstrating a heterogeneous prostatic mass, deemed to be causing bladder outlet obstruction (Figure 1).

**Figure 2.**
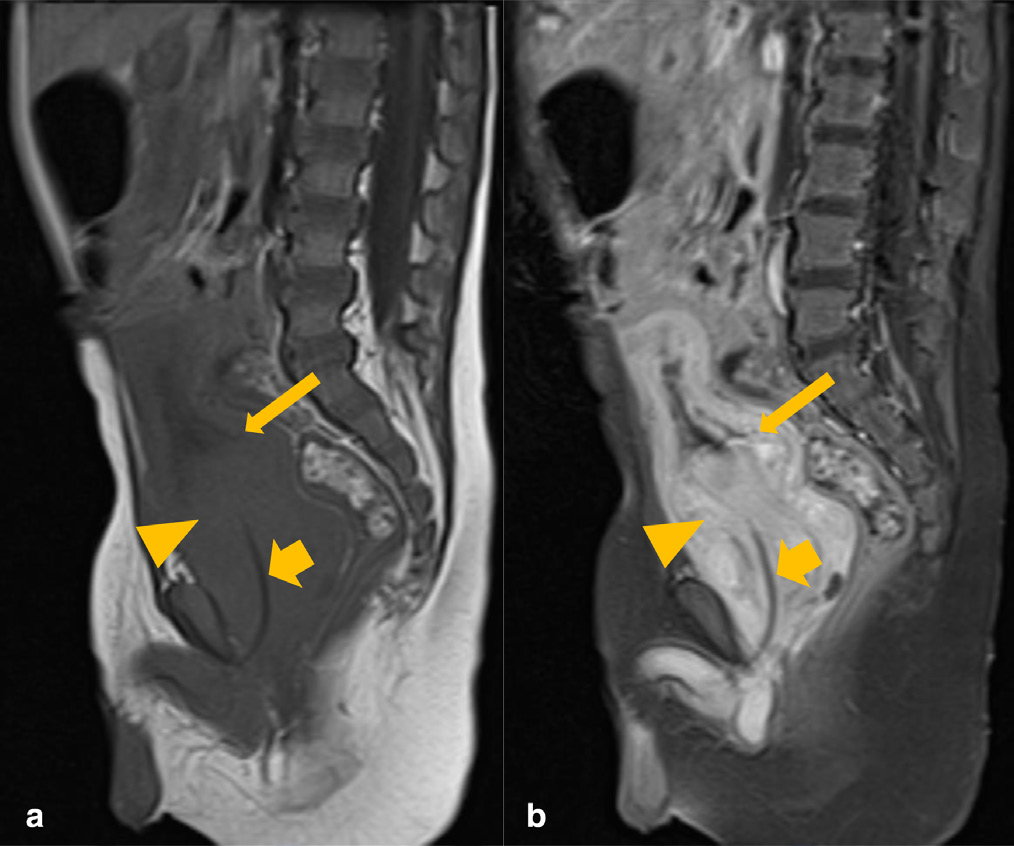
Sagittal T1 pre- (**A**) and post-contrast (**B**) MRI images of the abdomen and pelvis. There is an avidly enhancing prostatic mass (orange arrowhead), invaginating into the urinary bladder base (thin orange arrow). The urinary catheter is coursing through the mass (thick orange arrow).

These findings were further reinforced after an MRI abdomen and pelvis with contrast was performed (Figure 2).

The histological samples taken from the prostate gland confirmed morphological and immunohistochemical appearances in keeping with rhabdomyosarcoma.

**Figure 3.**
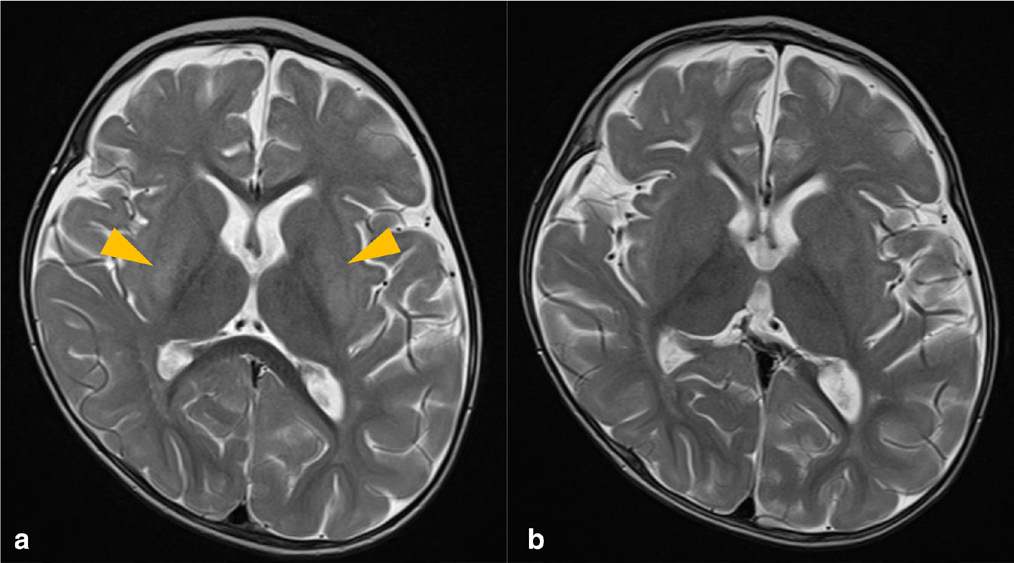
*T*_2_-weighted axial MRI sequences demonstrating high signal within the putamina bilaterally (orange arrowheads) (**A**), which has subsequently resolved upon follow-up imaging two months later (**B**).

The patient underwent an MRI head examination 7 days after admission (Figure 3), at which the renal function profile and neurological examination were within normal limits. The clinical indication was to exclude Neurofibromatosis type 1 (NF1), due to its known association with rhabdomyosarcoma. However, although this examination did not reveal any changes pertaining to NF1, it did reveal symmetrical high T2 signal change within the posterior putamina bilaterally, with no evidence of restricted diffusion or pathological post-contrast enhancement.

Follow-up imaging (Figure 3) 2 months after the initial presentation demonstrated resolution of these findings, correlating with normalised blood urea levels, suggestive of uremic encephalopathy (UE).

## Discussion

UE is an uncommon, acquired toxic metabolic disorder, associated with both acute and chronic renal failure. The cause is attributable to the accumulation of uremic toxins, such as creatinine and guanidine, which have the ability to activate the neurotoxic effect of NMDA receptors.^1^ UE has been well-documented in the adult population,^2^ but appears to be very rare in the paediatric population^3,4^. After a detailed literature review, to our knowledge, there are no case reports describing bilateral basal ganglia (BG) involvement in a non-diabetic, paediatric patient with no prior history of renal impairment.

The BG, the primary location of abnormality in this case, consist of the caudate nuclei, putamina and globus pallidi; complex structures that control extrapyramidal motor activity.^5^ The BG require a steady supply of adenosine triphosphate (ATP) to maintain transmembrane ion gradients, vital for their neuro-transmissive role. If the normal process of its generation, via aerobic respiration, is interrupted, as seen in conditions such as acute toxic metabolite accumulation, hypoxia, hypoglycaemia and carbon monoxide (CO) poisoning, then insufficient ATP production occurs via anaerobic mechanisms.^5^

UE has 3 principal patterns of imaging findings, best demonstrated as increased signal on T2 and FLAIR MRI sequences bilaterally, in keeping with vasogenic oedema from the accumulation of interstitial fluid, following a disruption of autoregulation.^2^ Firstly, the most common has cortical involvement, and is a category of posterior reversible encephalopathy syndrome ^6,7^ ; the second is rarer and demonstrates bilateral BG involvement^2^ ; and the final one has white matter involvement, limited to case reports.^6^ Very rarely, as described in a case report concerning a 14-year-old girl by Jia et al., there can be brainstem involvement.^3^ The ‘Lentiform Fork Sign ’ is characteristically associated with metabolic acidosis and blood-brain barrier disruption. It is demonstrated as a hyperintense T2 rim delineating the medial (internal capsule, internal and external medullary laminae) and lateral (external capsule) boundaries of the putamina bilaterally.^2^ Restricted diffusion, suggestive of cytotoxic oedema, can occur in UE, but is usually limited to the globus pallidi, as it is more vulnerable to cytotoxic damage than the putamina, due to higher ATP requirements.^2^ Post-contrast enhancement is also not a typical feature. In most cases, lesions on neuroimaging improve or resolve upon normalisation of uremia.^6^

In the acute setting, with bilateral symmetrical BG pathology, as demonstrated in this case, one must also consider causes such as hypoxia, hypoglycaemia, CO poisoning, haemolytic uremic syndrome and osmotic myelinolysis, in the presence of appropriate history and laboratory findings.^8^

In hypoxia, the acute deprivation of oxygen means anaerobic metabolism predominates. This results in the formation of lactic acid, which changes the intracellular environment, resulting in diffuse BG injury,^9^ and possibly associated haemorrhage.

Hypoglycaemia, as opposed to hypoxia, does not produce lactic acidosis as there is a different mechanism of injury. However, the endpoint of both processes, namely ATP depletion, is similar. The radiological findings include high T2 signal change bilaterally within the posterior limbs of the internal capsules, BG, cerebral cortex (parieto-occipital and insular) and hippocampi. CO poisoning impairs oxygen delivery to the BG, predominantly by competitive binding to haemoglobin.^10^ Interestingly, it has an affinity for globus pallidi injury, although the remaining BG may also be affected. Moreover, there is cerebral white matter injury, which in fact determines the overall prognosis.^10^

Haemolytic-uremic syndrome is a rare and multisystem disorder, with most patients presenting before the age of 5 years. It is characterised by microthrombosis, acute renal failure, thrombocytopenia and microangiopathic haemolytic anaemia. Vascular occlusion results in impaired delivery of both oxygen and glucose to the BG, leading to decreased ATP production and ultimately BG injury.^11^

Osmotic myelinolysis, which can occur following the rapid correction of sodium concentrations, typically affects the pons. However, the BG can be affected (extrapontine myelinolysis), with symmetrical high T2 signal change affecting the lentiform nuclei bilaterally.^12^ There was no history of rapid sodium correction in this case.

The key, non-acute differential diagnosis to consider with these imaging findings, is NF1 or von Recklinghausen disease, as there is an association with rhabdomyosarcoma.^13^ The mean age of cancer diagnosis, however, is 2.5 years, which is significantly older than our patient.^13^ Intracranial manifestations of NF1, specific to the BG, include focal areas of signal intensities (FASIs), which are focal areas of high T2 signal with no mass effect or contrast enhancement, representing myelin vacuolisation, which may wax and wane for the first decade of life.^14^ These lesions favour the globus pallidi^15^ and are usually not symmetrical, hence differing from the features seen in our case. Reversal of the signal changes in a relatively short time period, as seen in this case, is also unusual in NF1.

## Conclusion

This case highlights bilateral and symmetrical high putaminal T2 signal change in a 1-year-old boy with acute renal failure and hyperuremia secondary to prostatic rhabdomyosarcoma, resolving upon correction of the abnormal urea levels, thus suggestive of UE. Other acute causes of bilateral BG changes were considered, but these did not correlate with the clinical history or specific laboratory findings. Rhabdomyosarcoma is associated with NF1, but the MRI findings do not support this diagnosis.

## Key learning points

1. UE can be associated with the ‘Lentiform Fork Sign’ in MRI imaging.
2. The abnormal MRI findings resolve upon correction of the raised urea levels.
3. It is important to consider other acute causes of bilateral BG radiological abnormalities, including hypoxia, hypoglycemia, CO poisoning and osmotic myelinolysis.
